# Osteopontin Expression in the Brain Triggers Localized Inflammation and Cell Death When Immune Cells Are Activated by Pertussis Toxin

**DOI:** 10.1155/2014/358218

**Published:** 2014-11-24

**Authors:** Maria Cecilia Garibaldi Marcondes, Ryan Ojakian, Nikki Bortell, Claudia Flynn, Bruno Conti, Howard S. Fox

**Affiliations:** ^1^Molecular and Cellular Neuroscience Department, The Scripps Research Institute, 10550 North Torrey Pines Road, La Jolla, CA 92037, USA; ^2^Immunology and Microbial Science Department, The Scripps Research Institute, La Jolla, CA 92037, USA; ^3^Chemical Physiology Department, The Scripps Research Institute, La Jolla, CA 92037, USA; ^4^Department of Pharmacology and Experimental Neuroscience, University of Nebraska Medical Center, Omaha, NE 68198, USA

## Abstract

Upregulation of osteopontin (OPN) is a characteristic of central nervous system pathologies. However, the role of OPN in inflammation is still controversial, since it can both prevent cell death and induce the migration of potentially damaging inflammatory cells. To understand the role of OPN in inflammation and cell survival, we expressed OPN, utilizing an adenoviral vector, in the caudoputamen of mice deficient in OPN, using beta-galactosidase- (*β*-gal-) expressing vector as control. The tissue pathology and the expression of proinflammatory genes were compared in both treatments. Interestingly, inflammatory infiltrate was only found when the OPN-vector was combined with a peripheral treatment with pertussis toxin (Ptx), which activated peripheral cells to express the OPN receptor CD44v6. Relative to *β*-gal, OPN increased the levels of inflammatory markers, including IL13R*α*1, CXCR3, and CD40L. In Ptx-treated OPN KOs, apoptotic TUNEL+ cells surrounding the OPN expression site increased, compared to *β*-gal. Together, these results show that local OPN expression combined with a peripheral inflammatory stimulus, such as Ptx, may be implicated in the development of brain inflammation and induction of cell death, by driving a molecular pattern characteristic of cytotoxicity. These are characteristics of inflammatory pathologies of the CNS in which OPN upregulation is a hallmark.

## 1. Introduction

Several central nervous system (CNS) dysfunctions, such as in multiple sclerosis (MS), viral encephalitis, Parkinson's disease (PD), and others [1–8], are highly correlated with inflammation, Interferons (IFN) and IFN-mediated genes. In a previous study, using the nonhuman primate model of neuroAIDS, we examined the expression pattern of genes in the CNS in correlation with signs of inflammatory pathogenesis and cognitive dysfunction [2]. Among the 10 most upregulated genes, Osteopontin (OPN), also known as secreted phosphoprotein 1 (Spp1), raised our interest as it appeared in close association with the presence of infiltrating macrophages [2] and also because it has been identified in other CNS pathologies [1–8]. We have confirmed the ability of OPN to attract inflammatory cells expressing the OPN receptor CD44, in a mouse model of peripheral cell migration [9]. Interestingly, in the macaque model of neuroAIDS, brain-infiltrating macrophages express a variant of CD44, CD44v6, which is one of the major OPN receptors [10], and has been shown to be a potential biomarker of CNS inflammation [11]. In neuroAIDS, OPN is thought to participate in the accumulation of macrophages in the brain by preventing recirculation and protecting inflammatory cells from apoptosis [12]. However, the contrasting mechanisms by which OPN drives local inflammation in the brain, have been poorly explored. Here, we developed a mouse model to assess the ability of locally driven OPN to induce a proinflammatory environment and to investigate what proinflammatory requirements are influenced by OPN in the brain tissue.

It is known that OPN regulates both innate and adaptive immune responses in the CNS and elsewhere in the body [13]. It is an Arg-Gly-Asp- (RGD-) containing acidic glycoprotein, and one of the major products of activated macrophages [14], which helps these cells participate in cell attachment motility during inflammatory processes [15–17]. OPN interacts with CD44 on the cell surface in a RGD-independent fashion [18] to modulate primarily macrophage migration as well as activation [19–22].

In spite of multiple lines of evidence that associate OPN with brain encephalopathy and CNS inflammation, the role of this molecule remains controversial, as OPN has also been identified in tissue repair processes [23]. In addition, the cellular mechanisms and pathways regulated by OPN are poorly understood, and the understanding of these mechanisms is complicated by the complexity of the inflammatory environment in the context of various pathologies.

In order to further investigate the role of OPN in brain lesions and inflammatory pathogenesis, we developed an* in vivo* system by which OPN expression was locally driven using adenoviral delivery into the brain of OPN deficient animals. This system allowed the identification of initial molecular requirements driven by the local expression of OPN towards favoring inflammatory infiltration. These molecules offered insights into potential mechanisms of pathogenesis and neurotoxicity in CNS pathology that could be mediated by OPN.

## 2. Materials and Methods

### 2.1. Mice

OPN deficient mice (B6.129S6(Cg)-Spp^1tm1Blh^/J) were obtained from Jackson Laboratories (Bar Harbor, ME; stock #004936) and bred at The Scripps Research Institute. The genotype was confirmed by PCR analysis. All animals were housed in a specific pathogen-free facility with unlimited access to water and laboratory chow. The experiments were approved by the TSRI Institutional Animal Use and Care Committee of our institute and were conducted in accordance with the guidelines of the institutional animal care policy.

### 2.2. Adenovirus Constructs

The cDNA plasmids carrying the OPN or *β*-gal coding sequences were purchased from Open Biosystems, by Life Technologies (Carlsbad, CA, USA). The OPN sequence corresponds to the “a” allele found in B6 and BALB/c mice (GenBank accession number BC057858) [24]. Sequence and expression analysis were performed by cloning the cDNA into pCMV-SPORT6. Protein expression was achieved in HEK293. The expression of OPN was confirmed in supernatants by Western blot, using anti-mouse osteopontin (clone O-17, IBL, Japan). The sequenced DNA plasmid was used for the adenoviral vector construction, which was performed by Vector Biolabs (Philadelphia, PA, USA).

### 2.3. Injection

Anesthetized animals were injected 1 × 1 mm^2^ and 2.0 mm lateral from midline, into the area stereotactically defined as the caudoputamen, within the striatum dorsal region (reference p56, coronal). The injection site is at the midline between the base of the ear and the eye. The viruses were injected at 5 × 10^5^ pfu in 2 uL volume of sterile PBS containing 3% Evans Blue (Sigma Aldrich, St. Louis, MO, USA), to allow for localization of the injection site and to address reproducibility. Twenty-four hours later animals received 300 ng of Ptx dissolved in saline, or saline alone, intraperitoneally. After 6 days, brains were harvested from perfused animals. The contralateral lobe, as well as deep cervical lymph nodes, was used as within-animal controls. *β*-gal-injected animals were used as between-group controls.

### 2.4. Probe Development

To make a probe for* in situ* hybridization, a 410 bp sequence of OPN was amplified by PCR using primers 5′-TAGGGTCTAGGACTAGCTTG-3′ and 5′AATCGTCCCTACAGTCGATG-3′. The fragment was molecularly cloned into pCR 2.1 TOPO TA. The resulting plasmid was then digested with BamH and XhoI and subcloned into pBS SK+. Purified DNA was linearized with either XhoI (antisense) or BamH (sense), purified, and radioactively labeled for* in situ* hybridization as previously described [25].

### 2.5. *In Situ* Hybridization, Immunohistochemistry, Histology, and Apoptosis Quantification

After perfusing animals with PBS containing 5 mM of EDTA (Gibco Life Technologies, Grand Island, NY, USA), brains were divided into experimental (ipsilateral) and control (contralateral) hemispheres, which were fixed in 10% buffered formalin or Carnoy's fixative, followed by 70% ethanol. Tissues were embedded in paraffin, cut into 7 *μ*m sections, and mounted on glass slides. Rehydrated sections were stained with hematoxylin and eosin or subjected to either* in situ* hybridization or immunohistochemical staining procedures. For immunohistochemistry, endogenous peroxidase activity was blocked by a 3% hydrogen peroxide treatment in absolute methanol. Following that, a heat treatment with 0.01 M citrate pH 6.39 was performed for antigen exposure. Sections were blocked with 5 g/L Casein (Sigma Aldrich) in PBS, containing 0.5 g/L thimerosal (Sigma Aldrich) and incubated with the primary antibody diluted in the same buffer. Antibodies were targeted against F4/80 (eBioscience, San Diego, CA, USA), Iba-1 (Wako Chemicals, Richmond, VA, USA) and Mac-3 (eBioscience). Biotinylated secondary antibodies (goat anti-rabbit IgG or rat anti-goat IgG, Vector Labs, Burlingame, CA, USA) were used at 1/300 dilutions. Visualization was achieved using biotin/avidin-peroxidase (Vector Labs) and Nova Red (Vector Labs). Counterstaining was made with Gill's hematoxylin. For TUNEL staining, paraffin-embedded sections were labeled according to the* In Situ* Cell Death Detection Kit (Roche Applied Sciences, Indianopolis, IN, USA) protocol. Counting the number of TUNEL+ cells was performed using the Abercrombie correction factor [26–28], as follows. Stained 7 *μ*m sections, cut with intervals of 3 starting from where the lesion was first detected until its end (5–7 sections), were inspected at a 60x magnification. A counting frame defined the length and width perimeter of the lesion in both dimensions. Total cells in the section were also counted. The number of positive cells in the counting frame was determined using the formula *P* = *A*[(2*SM* − *L*)/*SM*(*S* + 1)], where *P* is the number of TUNEL+ positive nuclear points per section, *S* is the number of sections in which the average is maintained, *A* is the crude count of number of nuclei seen in the whole section, *M* is the thickness (in *μ*m) of the section (7 *μ*m), and *L* the average length (in *μ*m) of the nuclei (7 *μ*m).

For* in situ* hybridization, formalin-fixed, paraffin-embedded rehydrated sections were prepared as mentioned above, with heat-treatment in citrate buffer; then, sections were incubated for 1 hour at 42 to 46°C in a prehybridization buffer (50% formamide, 0.3 M NaCl, 20 mM Tris, pH 8, 5 mM ethylenediaminetetraacetic acid, 1 M Denhardt's solution, 10 mM dithiothreitol, and 10% dextran sulfate in diethyl pyrocarbonate-treated water) and hybridized with 3 × 10^6^ cpm radiolabeled probes, prepared as above, in the same buffer at 42 to 46°C overnight. Controls included sense probes. After hybridization, sections were washed, treated with RNase and stained with Iba-1 antibody, performed as described above except that the substrate development was made with HistoMark Orange (KPL, Gaithersburg, MD, USA). The slides were then washed, dehydrated, vacuum-dried, and coated with Kodak NTB2 emulsion (Eastman Kodak, Rochester, NY, USA). The slides were then left in the dark for 10 days before developing (Kodak D19) and fixing (Kodak “Fixer”) (Eastman Kodak, Rochester, NY, USA), followed by counterstaining with Methyl Green (Sigma Aldrich), dehydration with isopropanol, and mounting.

### 2.6. Brain Cell Suspensions

Individual brains were forced through a 70 *μ*m nylon sieve, and the disassociated material was collected by centrifugation at 470 ×g for 15 min at 4°C. The supernatants were aspirated, and each pellet was brought to a final volume of 10 mL with HBSS, after which 28 U/mL of DNase I and 500 U/mL of collagenase II were added. Digestion was performed at 37°C in a shaking water bath for 1 h. Afterward, the cells were pelleted at 470 ×g for 10 min and then washed in HBSS containing 1% FCS. The cell pellet was resuspended in 2.1 mL of a 1.122 g/mL Percoll (GE Healthcare Bio-Sciences, Pittsburgh, PA, USA) solution and brought to a final volume of 10 mL with HBSS, resulting in 1.033 g/mL Percoll. Three milliliters of 1.088 g/mL Percoll was underlayed, and this gradient was centrifuged at 1200 ×g for 20 min at 20°C. The cells at the 1.033/1.088 interface were collected, washed, and quantified in hemocytometer.

### 2.7. Detection of Infiltrating T Cells and Macrophages

Cells isolated as described were stained with 50 *μ*L mixtures of antibodies diluted according to a previous titration in staining buffer (HBSS with 2% FCS and 0.01% NaN_3_). The antibodies used were against CD11b (eBioscience, San Diego, CA, USA), CD4 (BD Biosciences, San Jose, CA, USA) and CD8 (BD Biosciences). Matching isotype controls (BD Biosciences) were also used. The cells were then processed through a FACScalibur flow cytometer (BD Biosciences) and analysis was performed with FlowJo 6.2.1 software (Tree Star, Ashland, OR, USA).

### 2.8. RNA Extraction and QRT-PCR for OPN and for Chemokine Detection

Total RNA was purified from a 2 mm^3^ section of caudoputamen, containing the site of OPN or *β*-gal vector injection, using TRIzol Reagent (Invitrogen, Carlsbad, CA, USA) following the protocol of the manufacturer, with an additional centrifugation step to remove cellular debris. RNA was further purified (RNeasy mini kit; Qiagen, Valencia, CA, USA), and the quantity of total RNA was assessed with a Nanodrop ND-1000 Spectrophotometer (Nanodrop; Wilmington, DE, USA). cDNA was obtained using RT2 First Strand cDNA kit (Qiagen) and an array of 84 genes involved in inflammatory responses, including chemokines and receptors (PAMM-022Z, Qiagen) (see Supplementary Figure 1 in Supplementary Material available online at http://dx.doi.org/10.1155/2014/358218 for all the molecules tested in addition to OPN). PCRs were performed in ABI 7900HT Fast Real Time PCR apparatus (Applied Biosystems, Grand Island, NY, USA).

### 2.9. Statistics

Two-way ANOVA, followed by Bonferroni's test, was performed using Prism Software (Graphpad software, San Diego, CA, USA). Comparisons in qRT-PCR measurements were performed using PCR Array Data Analysis Software (Qiagen). Sections were performed in the injected lobes of 6 animals per group. Flow cytometry experiments were performed in 6 animals per group. qRT-PCRs arrays were performed in two independent experiments with the dissected lesion sites of 6 animals per group and performed in triplicate. Statistical analysis included (a) a between-group comparison, taking OPN-injected lobes and *β*-gal-injected lobes into account, and (b) a within-group comparison, taking the vector-injected hemispheres and equivalent regions of contralateral hemispheres into account. The second was performed as a control for inoculum leaks, which greatly behaved as controls, and therefore were not shown.

## 3. Results

### 3.1. Localized Expression of OPN Attracts Peripherally Activated Inflammatory Cells

De novo expression of OPN was driven by an adenoviral vector in the basal ganglia caudoputamen region, which is highly relevant in CNS pathologies with cognitive consequences, such as neuroAIDS, and particularly for being connected to the dopaminergic-neuron-rich substantia nigra [29]. We have performed viral dose-response experiments, to identify the ideal inoculum that was able to induce long-lasting gene expression using the *β*-gal construct, and to further confirm results with the OPN-encoding vector. The dose of 5 × 10^5^ pfu was able to induce gene expression which was contained to the site of injection, as early as 3 days after delivery, with a peak on day 6, and that lasted up to 21 days after delivery (data not shown). The dose of 10^6^ pfu induced a similar expression pattern as the one we observed and described here (data not shown). Doses of 5 × 10^6^ and above were characterized by a short 2–4-day gene expression, both in the *β*-gal and in the OPN-injected brains, as detected by LacZ and* in situ* hybridization, respectively, and a not consistent increase of inflammatory cells. On the other hand, using doses that were lower than 5 × 10^5^ pfu, we were not able to consistently detect changes in gene expression between groups. The addition of 3% Evans Blue to all the inocula allowed the identification of the injection site, and also the detection of potential leaks to the periphery and to the contralateral hemisphere. In order to determine the volume of the inoculum, leaks were inspected in peripheral CNS draining lymph nodes (deep cervical, superficial cervical, nasal, and periaortic), 5 and 10 minutes after the injection in pilot experiments, and in the contralateral hemispheres. The latest were systematically assessed to identify not only leaks, but also potential broad effects resulting from a localized gene expression.

**Figure 1:**
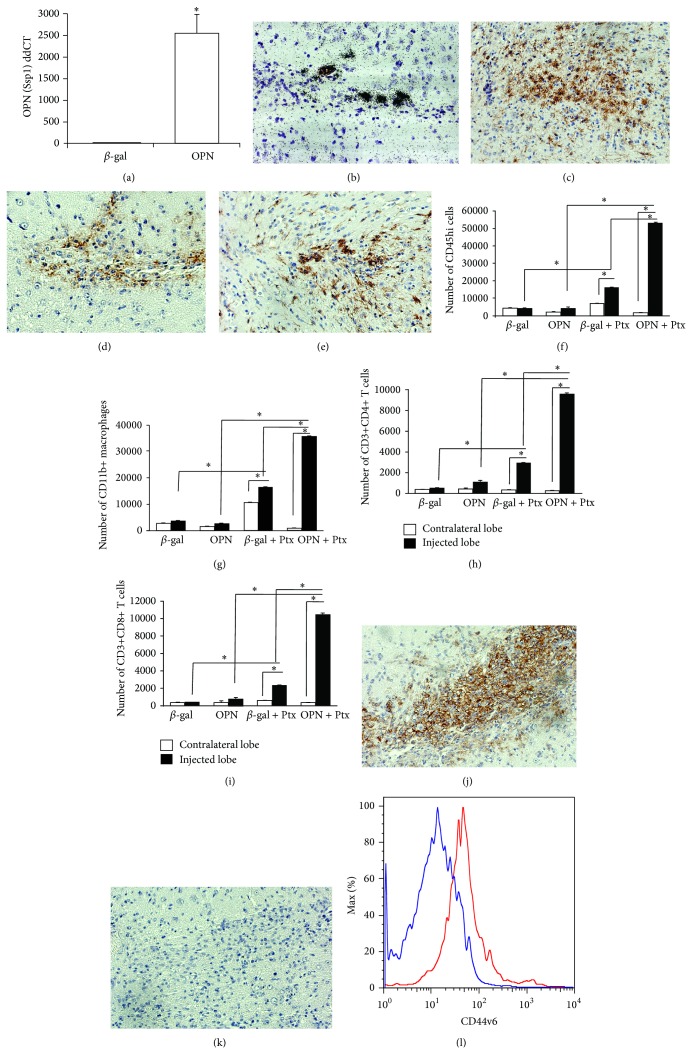
Induction of OPN in the brain promotes migration of peripherally activated leukocytes. The adenoviral vector encoding the OPN gene, injected between the cortex and the putamen, induced OPN (Spp1) expression that was detected by (a) qRT-PCR for OPN (Ssp1) detection in lesion fragment site. ^*^ *P* < 0.05, *t*-test. (b)* In situ* hybridization for detection of OPN expressed in a representative lesion site tissue, 6 days after injection. Animals were also injected with pertussis toxin (Ptx) ip. The brains injected with the OPN-vector or with a control adenovirus encoding *β*-gal (*β*-gal), were separated in contralateral lobe (white bars) and injected lobe (black bars and transformed in cell suspensions as described [74]. (c) Iba-1+ cells (brown) with morphological characteristics of activated microglia in a site of lesion of a representative OPN-injected mice, showing that microglia activation was highly restricted to the delivery site, as detected by immunohistochemistry on a serial section. (d) F4/80+ cells (macrophages—brown) in the delivery site, 6 days after injection with OPN on a serial section of representative OPN-injected mouse; (e) Iba-1+ cells in *β*-gal-injected brains. The immune cell content, excluding resident microglia, was evaluated by FACS using fluorescent labeled antibodies against the indicated surface markers. (f) Gated CD45 high migrating cells, and among them (g) CD11b+ macrophages, (h) CD3+CD4+, and (i) CD3+CD8+ lymphocytes. ^*^ *P* < 0.05, Bonferroni's post hoc test. (j) F4/80+ cells (brown) in animals that received the OPN vector and were also stimulated with Ptx. (k) Isotype control staining on a Ptx-stimulated OPN-injected mouse. (l) Histogram of CD44v6 fluorescence intensity, on CD11b-gated cells, upon FACS analysis of brain-draining deep cervical lymph nodes, 6 days after i.p. injection of saline or Ptx into animals that received the OPN vector. Blue line shows animals injected with saline, and the red line shows cells from Ptx-stimulated mice.

Injection of the recombinant adenoviral vector encoding OPN into the caudoputamen of OPN-deficient mice, based on a previously described model [30], led to a localized expression of OPN (Figures 1(a) and 1(b)), and was restricted to the injection site (Figure 1(b)); similarly, the *β*-gal containing vector led to the expression of *β*-gal (data not shown). The accumulation of inflammatory cells in the nonperipherally activated OPN-injected ipsilateral injected or contralateral hemispheres (to control for leaks), or in comparison with the *β*-gal injected brains, 6 days after injection, was not significantly different, as measured by the expression of CD45hi-gated cells by FACS, which characterize infiltrating leukocytes of peripheral origin (Figure 1(f)). Iba1+ cells with morphological characteristics of activated microglia were more abundant in the site of OPN injection (Figure 1(c)) when compared to the site of *β*-gal injection (Figure 1(e)), as detectable by IHC. F4/80+ macrophages were localized adjacent to the OPN-expressing lesion site (Figure 1(d)), as determined by IHC, and their numbers were not different in comparison to *β*-gal-injected sites (Figure 1(g)). We also observed a local increase of GFAP+ astrocytes (not shown).

The* Bordetella pertussis* toxin or pertussis toxin (Ptx) is known to increase the blood-brain barrier (BBB) permeability [31, 32], and facilitate immune cell migration into the CNS [33, 34]. It has been used in a model of adenoviral delivery of IFN*γ*, working as a surrogate for environmental conditions that trigger CNS inflammatory pathologies [35]. Historically, Ptx has been used as an adjuvant for the induction of experimental autoimmune encephalomyelitis (EAE) since 1955 [36]. Therefore, we followed the CNS injection of adenoviral vectors with a peripheral, intraperitoneal injection of Ptx (or saline as control). Inflammation was quantified by isolation of cells from the ipsilateral (injected) or contralateral (noninjected) hemispheres (to control for leaks and the effect of Ptx within animals), and by comparing OPN-injected animals with *β*-gal-injected controls. Quantification was performed based on the surface identification of immune markers and FACS analysis.

In animals stimulated with Ptx, the peak of cell infiltration occurred 6 days after infection, in correlation with OPN expression, and was followed by a gradual decay of the number of infiltrating cells. The characterization of the infiltrating cells by FACS, at 6 days, revealed a significant increase of CD45hi cells in the ipsilateral lobe of Ptx-injected animals, both in *β*-gal and in OPN-injected brains, but was especially marked in the OPN mice relative to the *β*-gal (Figure 1(f), *P* < 0.0001, 2-way ANOVA followed by Bonferroni's test). Importantly, the examination of the contralateral naïve hemisphere of the brains revealed that the focal expression of viral vector encoded products did not affect the BBB in a broad manner. Taking into account the injected lobes, the CD45hi-gated infiltrating immune cells in OPN-injected animals were predominantly CD11b+ (also CD11c-Gr1^low^) macrophages (Figure 1(g)), but cells expressing T cell markers, CD3+CD4+ (Figure 1(h)) and CD3+CD8+ (Figure 1(i)), were also identified. The enrichment of macrophages in the brains of mice receiving the OPN vector and Ptx treatment was confirmed by immunohistochemistry for the detection of F4/80+ macrophages in the site of lesion (Figure 1(j)). Isotype controls were performed (Figure 1(k)). Cells with morphology of neutrophils or expressing CD11b+ Gr1high were not observed in the injection site. Many mechanisms for Ptx action have been proposed, such as increased BBB permeability and stimulation of leukocyte infiltration [31, 32, 34]. We hypothesized that Ptx-injected animals increased the expression of OPN receptor in peripheral immune cells, particularly in CD11b+ macrophages. Indeed, using flow cytometry on cells isolated from the peripheral sites, such as brain-draining deep cervical lymph nodes, we found that gated CD11b+ CD11c-Gr1low macrophages from Ptx-injected animals (red line) had increased expression of the CD44v6 isoform, when compared to animals injected with saline into the peritoneum (blue line) (Figure 1(l)).

### 3.2. OPN Drives a Molecular Expression Pattern That Characterizes Neurotoxicity in Other Models

**Figure 2:**
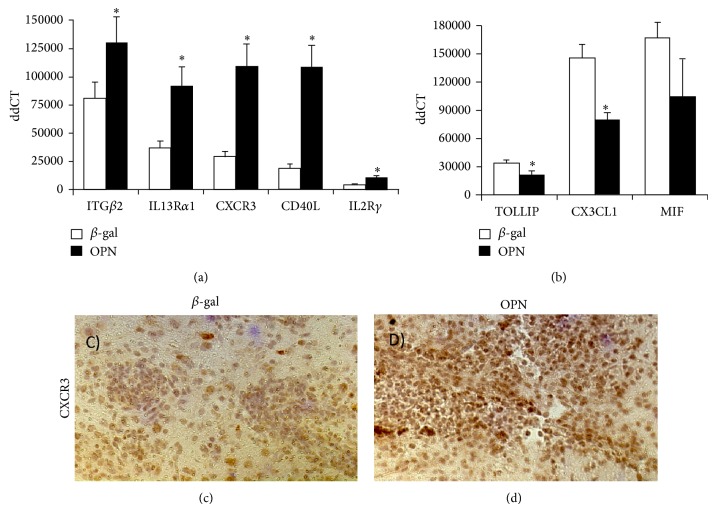
Proinflammatory molecules modified by OPN. QRT-PCR was used to evaluate levels of chemokine receptors and proinflammatory molecules using SABiosciences PCR array, and SyBr Green/ROX detectors in an ABI HT7900 Fast apparatus. We measured 84 genes involved in inflammatory responses, including chemokines and receptors (PAMM-022Z, Qiagen). Results show the genes that were significantly changed. (a) Genes upregulated in OPN-injected brain lobes compared to *β*-gal controls. (b) Genes downregulated in OPN compared to *β*-gal controls. Results represent average ± SD of 6 experimental lobes injected with either *β*-gal or OPN-encoding vector. Experiments were performed in triplicate. ^*^ *P* < 0.05 (*t*-test) in comparison to *β*-gal. Immunohistochemistry was utilized for validation. (c) Immunohistochemistry for tissue detection of CXCR3 in representative sections of the vector injection site, in *β*-gal and Ptx group, and in (d) OPN-encoding vector and Ptx.

To assess the OPN-induced factors that may contribute to the infiltration of immune cells from the stand point of OPN expressing cells, we performed qRT-PCR to inspect the expression of various transcripts of inflammatory molecules for potential change with localized OPN expression, using animals that were not stimulated peripherally with Ptx (Table 1), in order to eliminate transcripts from such infiltrating cells. The selection of genes was performed based on their described importance for cell migration to inflammatory sites (See Supplementary Figure for a complete list of molecules analyzed and their expression levels). We have identified genes that were significantly changed at 6 days after injection (using a fold-change of >|2| and uncorrected *P* < 0.05) in OPN versus *β*-gal (Figure 2). There was a significant 2.3-fold increase in the expression of IL13R*α*1 (*P* = 0.0082), a 2.4-fold increase in CXCR3 (*P* = 0.015), a 3.38-fold increase in CD40L (*P* = 0.03), and a 2.4-fold increase in IL2R common gamma chain (*P* = 0.02) and OPN itself (*P* < 0.001) (Figure 2(a) and Table 1). We also observed a 0.8-fold downmodulation of TOLLIP (*P* = 0.03) and a 0.5-fold down modulation of CX3CL1 (*P* = 0.04) (Figure 2(b)). A substantial but not significant 0.6-fold decrease in MIF was also noted (Figure 2(b)). In OPN-injected animals that were peripherally stimulated with Ptx, we also observed an upregulation of CXCR3 (Figures 2(c) and 2(d)) at the lesion sites, compared to *β*-gal, as detected by immunohistochemistry.

**Figure 3:**
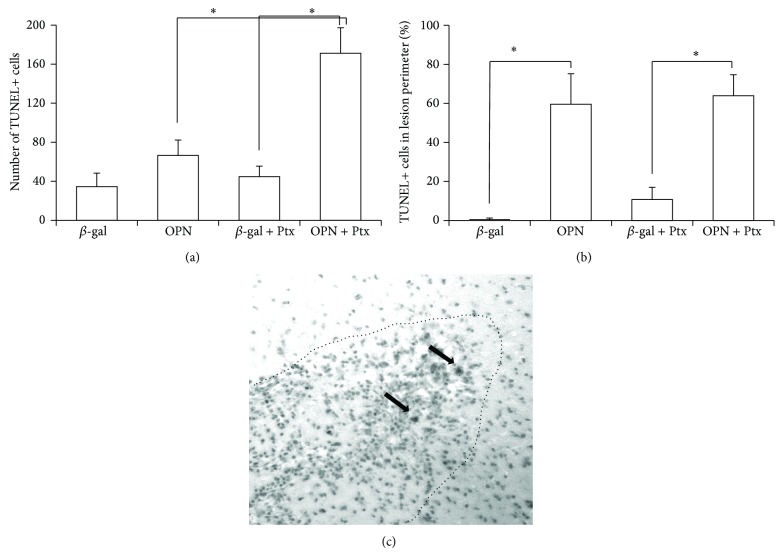
Number of TUNEL-positive cells in brain sections after the injection adenoviral vector constructs. The number of TUNEL-labelled apoptotic cells was counted under microscope in 5 sections from each of 6 animals/group, 21 days after the injection of *β*-gal or OPN constructs. The total number of TUNEL+ cells/lobe was estimated by applying the Abercrombie correction factor. (a) Total number of TUNEL+ cells in the brain lobe injected with the adenoviral vector bearing *β*-gal or OPN gene, in OPN−/− animals that were previously treated or not with i.p. Ptx. (b) Percentage of TUNEL+ cells identified within the focal lesion. ^*^ *P* < 0.05, ANOVA followed by Bonferroni's post hoc test, to include contralateral lobe putamen sections. (c) Representative section of the brain of OPN + Ptx animal, at the injection site, showing the lesion perimeter (dotted line) and TUNEL+ cells (black arrows) both within and outside the lesion limits.

Since both the molecular profile induced by OPN and the inflammation induced locally by the peripheral treatment with Ptx can be associated with neuronal damage and cell death, we next examined whether there were differences in the number of apoptotic cell bodies in the brain surrounding the vector injection site, as well as outside of the defined lesion perimeter (Figure 3(c)). In animals that received *β*-gal, either with or without Ptx treatment, apoptosis was not detectable near the infection site (Figures 3(a) and 3(b)). In contrast, OPN-injected animals had an increase in TUNEL+ cells by the lesion, especially upon peripheral stimulation with Ptx (Figures 3(a) and 3(b)). The identification of the dying cells was compromised by the strong background of picnotic bodies (not shown), interestingly, though, about 50% of the TUNEL+ cells were concentrated within the lesion perimeter (Figure 3(b)), suggesting that they could be either neurons or inflammatory cells. Figure 3(c) shows a representative section of a Ptx-stimulated, OPN-encoding virus injected brain, demonstrating the lesion perimeter and the presence of TUNEL+ cells.

## 4. Discussion

The adenoviral vector encoding the OPN gene injected into the brain of OPN deficient mice is a clean system to allow the identification of characteristics of CNS pathogenesis that are specifically driven by the reinsertion of OPN and its de novo expression. OPN was locally driven in the brain in animals that lack endogenous OPN, in order to specifically characterize the role of a local upregulation, detected both by* in situ* hybridization and by PCR. We did not examine the translational capacity of the OPN gene encoded by the adenoviral vector, regarding protein levels and biochemical integrity. However, the delivery of the OPN gene locally in the brain seemed to be physiological (as* per se*, it did not induce inflammation in nonperipherally activated animals), but sufficient enough to cause changes in the CNS microenvironment that were distinct from *β*-gal-injected controls. Furthermore, these changes induced by OPN expression were able to synergize with the peripheral immune activation provided by the injection of Ptx to induce significant inflammation and cell death. These two main components, OPN and peripheral activation of innate immune cells by Ptx, cooperate to induce an inflammatory infiltrate characterized mostly by macrophages and apoptosis. In addition, the molecular pattern generated by the expression of OPN was associated to neuronal damage and death. The induction of an inflammatory scenario by Ptx, following the induction of OPN, resembled in many aspects the results from Millward et al. [35], who showed that infiltration into the brain could also be achieved by the combination of Ptx and the viral delivery of IFN*γ*. In addition to inducing CD44v6 on infiltrating cells, Ptx in combination with OPN and its associated molecular pattern could also facilitate cell migration by inducing a BBB disruption, similar to what has been described in combination with an overexpression of MCP-1 or IFN*γ* [35, 37], through the induction of metalloproteinases [38].

In response to OPN in the brain, one of the upregulated genes was IL13R*α*1, which can play an important role in mechanisms of cytotoxic neuronal loss, as described by us [39]. The ligands of the IL13R*α*1, IL13 and IL4, are components of type II immune responses, which characterize allergic reactions and helminth infections [40–42], and, interestingly, are also involved in the susceptibility of DA neurons to death by oxidative stress [39]. Constitutively in the brain, dopaminergic (DA) neurons are the main cells that express this receptor, which is a contributing factor in the selective loss of DA neurons in regions of the substantia nigra (SN), mimicking a phenotype observed in PD [39]. Others have also suggested a link between OPN upregulation, inflammation, and DA neuronal loss [43].

We assessed whether the increase in OPN levels in the brain tissue can trigger a molecular pattern that is able to favor the accumulation of inflammatory cells. We found that the ability of OPN to induce inflammation happened in correlation with its ability to upregulate CXCR3 (the receptor for the chemokines CXCL9, CXCL10, and CXCL11) and CD40L. Specifically, in addition to its chemoattractant properties on T cells, in the brain, CXCR3 expression is able to mobilize microglia cells and contribute to dendrite loss [44]. CD40L has been found to synergize with other pathogenic molecules to result in neuronal injury and death [45, 46]. In addition, CD40L may also be involved in neurodegeneration through an oxidative stress-mediated mechanism [47, 48], and in BBB disruption [49]. Therefore, these molecules may be key prerequisites for inflammatory cell migration. However, in order to actually induce the migration of peripheral CD45hi macrophages and T cells, a peripheral trigger for the activation of these cells by Ptx was imperative to upregulate CD44v6 in this model and to likely induce other ligands that may facilitate BBB crossing, as previously suggested by other models [11].

We also observed that TOLLIP and CX3CL1 were downmodulated by OPN. Conversely, such molecules have been described to have an effect on neuronal survival. For instance, TOLLIP is an inhibitor of TLR signaling pathways [50], and its overexpression was proposed to protect neurons from toxic protein misfolding [51, 52]. This suggests a role for OPN, by inducing the decrease in TOLLIP, as an enhancer of TLR-mediated responses. CX3CL1 is expressed by microglia [53], where it reduces toxicity and, consequently, neuronal damage [54–59]. However, in models of transient cerebral ischemia and Alzheimer's disease (AD), CX3CL1 is reported to play opposite roles [60, 61].

Importantly, brains injected with the OPN-encoding vector had more cells expressing cell death markers in comparison with brains injected with *β*-gal, which were restricted to the lesion site. The induction of cell death induced by OPN has been reported in other models [62, 63]. This is in contrast with reports of a protective role played by OPN against apoptosis, including in the CNS [12, 63–66]. The accumulation of inflammatory cells in OPN-rich sites can be also a result of OPN's functional ability to prevent cellular regress from the CNS back to the circulation, in the so-called reverse transmigration across the endothelium [12]. However, it is important to notice that we have used OPN-deficient animals, who may be more susceptible to apoptosis by lacking this molecule endogenously. If so, we may assume that the cells suffering apoptosis are not cells that were infected by the OPN-encoding adenoviral vector. Indeed, in OPN-sufficient siblings, expressing the same background, similar experiments did not result in significant cell death, likely due to the protective effects of endogenous OPN (data not shown). In addition, given that TUNEL+ cells were especially enriched in areas that were within the inflammatory lesion, it is possible that other cell types are affected by the OPN-triggered molecular pattern.

The determination of the apoptotic cell types is a technical challenge, as picnotic cells were labeled with all antibodies. Thus, infiltrating inflammatory cells, local glial cells, or GABAergic neurons (given the localization of the lesion) can be potential targets of the cell death process under control of endogenous OPN. It is also necessary to highlight the fact that the peripheral stimulation with Ptx can play a synergistic role in the control of activation and cell death in the localized OPN-expressing sites. Importantly, it has been reported that the RGD-containing moiety of OPN is protective against neuronal loss in the context of inflammation [67]. This protective effect may be mediated by interactions between the exposure of the RGD binding domain of OPN, which can be achieved by the contact with thrombin, and integrin receptors, resulting in an attenuation of reactive gliosis. However, while the cleaved form is beneficial for neuronal survival, it is also present in brain tumors as a factor of cell survival and perpetuation [68]. Studies* in vitro* may be a key to determine the relative susceptibility of different brain cell subpopulations to cell death in the context of presence or absence of endogenous OPN, and exogenous active and cleaved forms, in addition to determining the role of peripheral activation as a requirement for cells to cross the BBB and cause pathology in the brain.

In physiological conditions, OPN has been found to be physiologically expressed by DA neurons and other neuronal populations in the basal ganglia, particularly in the substantia nigra, and absent in microglia and astrocytes [69]. However, in rodents, upon inflammatory stimulation with bacterial lipopolysaccharide, activated glial cells cause a local increase in OPN levels [70]. Therefore, in OPN-sufficient conditions, the local increase of OPN is likely a result of glial activation, and an overall proinflammatory environment. On the other hand, in a MPTP-induced PD model in marmosets, OPN, which is expressed exclusively by non-DA neurons, showed decreased levels following the treatment with the toxin, in spite of the increased gliosis [71]. A similar decrease of OPN levels in the basal ganglia has been described in human subjects with PD, multiple system atrophy and in progressive supracellular palsy [71]. The decrease in OPN in these models and conditions could be a result of loss of OPN-expressing subsets in a context of predominance of resident microglia over OPN-rich infiltrating macrophages [72]. Regardless, these results suggest that the mechanisms that lead to the loss of specific neuronal populations are not restricted to the expression of OPN and its ability to induce a neurotoxic molecular pattern, but that these mechanisms may influence outcome in some infections and inflammatory conditions that cause glial cells to strongly increase OPN or where infiltrating cells increase its levels locally.

A neurotoxic potential of OPN may depend on the presence of the inflammatory infiltrate that results from the combination of a peripheral activation of immune cells, the phenotypic pattern induced by specific peripheral stimuli, particularly regarding the upregulation of OPN receptors, and the upregulation of OPN in the CNS [11, 22, 73]. In our model, the expression of CD44v6 was induced by the peripheral stimulation with Ptx. Over all, the induction of OPN in the brain caused a molecular pattern that can be associated with a role in neurotoxicity. Our results indicate that OPN is an inflammatory component with a role in neurotoxicity and cytotoxicity. Strategies to target an upregulated expression of OPN in the CNS, as it is observed in neuropathology such as MS, PD, and neuroAIDS, without affecting endogenous levels, may be helpful to prevent neuronal loss. Further studies are necessary to individually define a role for OPN-mediated molecules in neuronal death. In summary, our study suggests that the localized expression of OPN triggers pathways potentially associated to cytotoxicity with consequences that are enhanced by the accumulation of inflammatory cells in the CNS, in a context of peripheral activation.

## 5. Conclusions

OPN is a proinflammatory inflammatory molecule that when expressed in the brain triggers molecules that are involved in neurotoxicity in various models. Therefore, the molecular microenvironment that is developed following a de novo expression of OPN can affect the viability of neurons. On the other hand, endogenous expression of OPN is a factor that can potentially rescue cytotoxicity. The role of specific cell types expressing OPN at endogenous levels or of its local upregulation for controlling neurotoxicity remains to be identified. Our model reveals that OPN is a balance component, with relevance in CNS pathologies such as MS, PD, and neuroAIDS, characterized by inflammatory infiltrate and upregulation of OPN levels.

## Supplementary Material

Total Transcripts measured in brain lesions from *β*-gal and OPN-injected brains. QRT-PCR was used to evaluate levels of chemokine receptors and pro-inflammatory molecules using SABiosciences PCR array, and SyBr Green/ROX detectors in an ABI HT7900 Fast apparatus. We measured 84 genes involved in inflammatory responses, including chemokines and receptors (PAMM-022Z, Qiagen), in the lesion segment of OPN -/- brains injected with b-gal or OPN-encoding Adv. Results represent the average ± SD of ddCT performed in a total of 6 animals per group, in triplicate. ∗p<0.05, ANOVA followed by Bonferroni's posthoc test.

## Figures and Tables

**Table 1:** Gene transcripts that were significantly changed in the lesion site following the injection with OPN-encoding adenoviral vector, in comparison to *β*-gal.

Gene name	Gene symbol	Fold change OPN/*β*-gal	*P* value
Secreted phosphoprotein 1	SPP1 (OPN)	505.54	0
Interleukin 13 receptor, alpha 1	IL13R*α*1	2.29	0.0083
Chemokine (C-X-C motif) receptor 3	CXCR3	2.39	0.0153
Toll interacting protein	TOLLIP	0.75	0.0178
Interleukin 2 receptor, gamma chain	IL2R*γ*	2.37	0.0237
Integrin beta 2	ITG*β*2	1.41	0.0329
CD40 ligand	CD40L	3.39	0.0348
Chemokine (C-X3-C motif) ligand 1	CX3CL1 (Fraktalkine)	0.55	0.0374

Fold change and *P* value of gene expression induced by OPN-in relation to *β*-gal-injected sites were calculated from qRT-PCR data, which was obtained from the examination of 84 genes assembled in a commercially available qPCR array, as described in Material and Methods. The genes are sorted according to significance.
