# Supplementary figures and images for: Osteopontin Expression in the Brain Triggers Localized Inflammation and Cell Death When Immune Cells Are Activated by Pertussis Toxin
Source: Mediators Inflamm. 2014 Nov 24;2014:358218. doi: 10.1155/2014/358218 (PMC4265371; doi:10.1155/2014/358218)

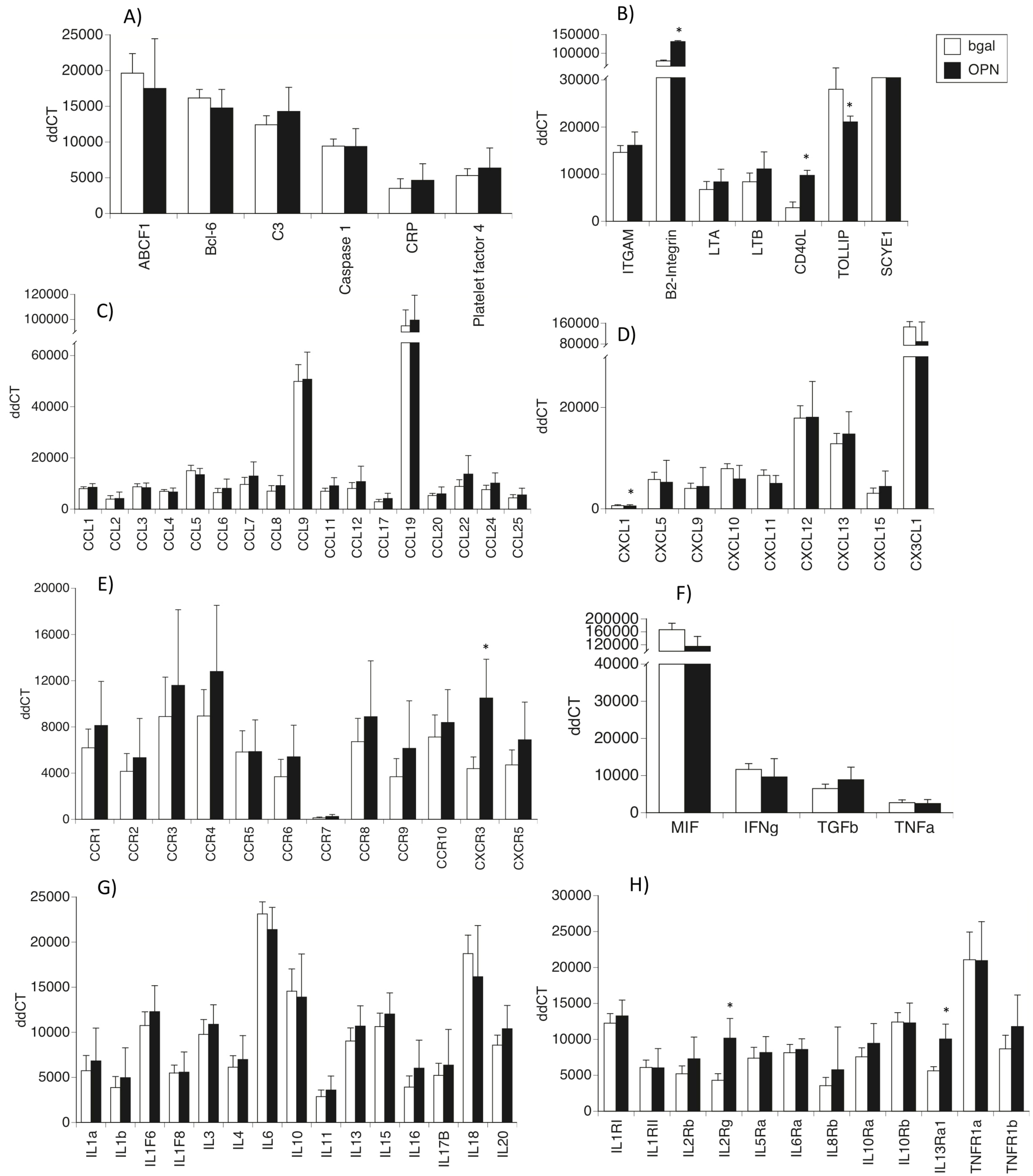

Supplement: Supplementary file 1 — Total Transcripts measured in brain lesions from β-gal and OPN-injected brains. QRT-PCR was used to evaluate levels of chemokine receptors and pro-inflammatory molecules using SABiosciences PCR array, and SyBr Green/ROX detectors in an ABI HT7900 Fast apparatus. We measured 84 genes involved in inflammatory responses, including chemokines and receptors (PAMM-022Z, Qiagen), in the lesion segment of OPN -/- brains injected with b-gal or OPN-encoding Adv. Results represent the average ± SD of ddCT performed in a total of 6 animals per group, in triplicate. ∗p<0.05, ANOVA followed by Bonferroni's posthoc test. [file 358218.f1.pdf]
